# Does health intervention improve socioeconomic inequalities of neonatal, infant and child mortality? Evidence from Matlab, Bangladesh

**DOI:** 10.1186/1475-9276-6-4

**Published:** 2007-06-05

**Authors:** Abdur Razzaque, Peter Kim Streatfield, Dave R Gwatkin

**Affiliations:** 1 Public Health Sciences Division, International Centre for Diarrhoeal Disease Research, Bangladesh; 2 Demography and Sociology Program, Australian National University, Canberra, Australia; 3 World Bank, Washington, USA

## Abstract

**Background:**

Although there are wide variations in mortality between developed and developing countries, socioeconomic inequalities in health exist in both the societies. The study examined socioeconomic inequalities of neonatal, infant and child mortality using data from the Matlab Health and Demographic Surveillance System of the International Centre for Diarrhoeal Disease Research, Bangladesh (ICDDR,B).

**Methods:**

Four birth cohorts (1983–85, 1988–90, 1993–95, 1998–00) were followed for five years for death and out-migration in two adjacent areas (ICDDR,B-service and government-service) with similar socioeconomic but differ health services. Based on asset quintiles, inequality was measured through both poor-rich ratio and concentration index.

**Results:**

The study found that the socioeconomic inequalities of neonatal, infant and under-five mortality increased over time in both the ICDDR,B-service and government-service areas but it declined substantially for 1–4 years in the ICDDR,B- service area.

**Conclusion:**

The study concluded that usual health intervention programs (non-targeted) do not reduce poor-rich gap, rather the gap increases initially but might decrease in long run if the program is very intensive.

## Background

Although a remarkable decline in mortality has been observed over last half of the past century, but within country, mortality both in developed and developing countries varied often by different sub-group [1,2]. Almost everywhere the poor suffer poor health and the gap in health condition by economic group, ethnicity, caste or place of residence remains very wide. As a consequence, the issue of health inequity, defined as 'inequalities in health status, risk factors, or health service utilization between individuals or groups, that are unnecessary, avoidable, and unfair' [3], has emerged as a major concern in the health field of the new century [1].

In Bangladesh, many positive changes have taken place in various fields (for example, in food production, communication, education, life expectancy, fertility decline) over the past few decades [4], but the country still remains one of the world's poorest nations according to World Bank criteria. To improve health of the people, the government has intensified health services over the country since the Alma-Ata conference in 1978. This includes establishment of Health Complexes and provision of free health and family planning services in both the urban and rural areas. In Matlab area, ICDDR,B has been maintaining a Maternal Child Health and Family Planning Program (MCH-FP) in half of the DSS area (ICDDR,B-service) since 1977, while other half of the area is treated as a comparison area (govt-service) where the government health and family planning program is in operation.

Earlier analyses of Matlab data documented that the poor-rich ratio of under-five mortality increased over time in both the areas but reduced slightly for 1–4 years in the ICDDR,B-service area [5]. The present study examined the issue further by adding two more birth cohorts with longer observation period along with more breakdown of age. The study emphasized whether health intervention programs reduced socioeconomic inequalities of neonatal, infant and child mortality and whether the inequalities have changed overtime.

### Health inequalities in developing countries

Although there are wide variations in mortality between developed and developing countries, socioeconomic inequalities in health exist in both the societies. The evidence shows not only that all countries have socioeconomic differentials in health, the magnitude and nature of these inequalities vary from place to place and time to time.

Lower mortality levels among children with higher parental socioeconomic status, often measured by occupation and education, have been observed in many developing countries [6-8]. In Bangladesh, household economic status as measured by ownership of various household articles had found negative relationship with child mortality [9]. Using data from Matlab area [10], a negative relationship was documented between mortality of children aged 1–4 and various socioeconomic factors.

Higher survival prospects for female children in industrial countries are nearly universal but exceptions to this pattern are observed for a number of developing countries [11]. For rural Bangladesh, higher female mortality compared to male after the neonatal period was reported consistently [12,13]. An examination of cumulative life-table probabilities of mortality by sex of the children for the cohort born during 1973–75 in Matlab area revealed that in ages 1–4, mortality for females exceeded that for males by 59 per cent [14]. A study conducted in Matlab area documented discrimination against female children in intra family food distribution and medical care; this was put forward as a likely mechanism for sex bias child health and survival [15].

Higher mortality rates in rural than urban areas have been documented in many developing countries [6]. Based on Bangladesh Fertility Survey data, higher mortality among rural children was reported by some studies [9,12]. After reviewing the relevant factors, it was identified that better medical, environmental, and socioeconomic conditions in the urban areas having a favorable effect [9].

Availability of better toilet facility was found to exert a positive influence on child mortality in many countries [16]. A study in a rural area of Bangladesh reported a positive association of use of latrine and a negative association of household size with survival during the post-neonatal period [17]. For childhood mortality an inverse relationship between dwelling area and use of fixed latrine was also observed in Matlab area [10].

### Health intervention and health inequalities

Studies showing the effect of health intervention on reducing socioeconomic inequalities in mortality are few, however, results are also not conclusive.

In Costa Rica, it was documented that the socioeconomic differentials of infant mortality narrowed when targeted primary health care services improved [18] while a similar pattern was reported in a cross-national study that childhood mortality by mother's education narrowed with the increase in per capita health expenditure [19]. In Colombia, it was found that increased access to health services disproportionately improved chances of child survival among less educated mother [20].

Based on Matlab data, although it was reported that the health intervention program had a greater effect on the risks of child death (1–4 years) of uneducated mothers than mothers with at some education [21] but re-analysis of the same data set did not confirm the result^a ^[Appendix A; [22]]. Again, it was reported that the poor-rich difference of 1–4 years mortality had decreased over time in the intervention area [23] while it was reported that measles vaccination narrowed the poor-rich difference of mortality of aged 9–60 months [24]. In another study, the impact of a woman-focused development programme on child survival was examined and found that there has been a 52% reduction of the pre-intervention level hazard of death of children during infancy of participant mother's compared to 31% reduction for the infants of on-participant mothers but during childhood (1–4 years) such reduction was similar in the two groups [25]. It was reported earlier that the poor-rich ratio of under-five and infant mortality had increased overtime but poor-rich ratio of 1–4 years mortality declined slightly in the intervention area [5].

Based on Nigerian data, it was reported that presence of modern health facilities had widened the childhood mortality by mother's education [26]. They argued that the educated mother has more knowledge of the modern world and enough self-confidence to secure urgent help for her sick children from medical professional. In Brazil, it was reported that the new interventions tend to increase inequity since they initially reach those who are already better off, at least until the wealthy reach a level beyond which little progress can be made [27].

## Data and methodology

### Setting

Data for this study come from Matlab *Upazila *(sub-district) where the International Centre for Diarrhoeal Disease Research, Bangladesh (ICDDR,B) has been maintaining a field station since 1963. Matlab is a rural area located about 55 km South-east of Dhaka. The area is low-lying deltaic plain intersected by the tidal river Gumti and its numerous canals. The major modes of transport within the area are walking, country boat and in some cases small steamer or launch. Farming is the dominant occupation, except in a few villages where fishing is the means of livelihood [28]. Most of the farmers are in marginal situations with less than two acres of land, and 40% of them are landless. For many families sharecropping and work on others' land on a daily wage basis have become the main sources of livelihood. Some people also work in mills and factories in different towns and cities but their families live in the study area. Rice constitutes the staple food and is harvested three times annually. Rates of illiteracy are high and increase with age.

In Matlab study area the ICDDR,B has been maintaining a Demographic Surveillance System (DSS) over 200,000 people since 1966. The DSS collects information on births, deaths, migration, marriages, divorce and household splits. The DSS events are collected by the Community Health Research Worker (CHRW) through monthly household visits and Field Research Supervisor (in the past, CHRW recorded events through fortnightly household visits and FRS accompanied by the CHRW visited the household every six weeks to complete the registration form). The DSS also maintains cross-sectional socioeconomic data and such data is available for 1974, 1982 and 1996.

The Matlab HDSS (DSS re-named as HDSS after integrating with the health data) area consists of ICDDR,B-service and govt-service areas. These two areas are almost similar in socioeconomic conditions [28] but differ in MCH-FP program [29]. Most of the ICDDR,B-service area was exposed to a contraceptive distribution program during 1975–77 and has been exposed to the Maternal Child health and Family Planning services since October 1977 [30]. In the ICDDR,B-service area, MCH-FP services have been provided by the CHRWs through fortnightly home visits until December 1999 and then from fixed site clinics. In fact, the intervention began with the family planning services and all other health services have gradually been added over a period of time. The CHRWs provide contraceptives and basic medicines to mother and child and referred patients with complications to sub-centre clinics. In addition, there are four sub-centres that also provide MCH-FP services. Moreover, ICDDR,B has a free 60-bed diarrhoea treatment centre in Matlab town and the facility is used not only by Matlab people but also from the surrounding districts. These health services are: tetanus toxoid vaccination to mothers; measles, DPT and polio vaccination to all children (by mid-1986 all blocks were covered); diarrhoeal disease management by *bari *mothers; vitamin A and beta-carotene supplementation; maternity care and antenatal check-ups; Acute Lower Respiratory Infection (ALRI) detection and management with penicillin and oral cotrimoxazole; education intervention to reduce ALRI mortality; reproductive tract infections detection and management; community based safe motherhood; and health centre assisted deliveries.

Until mid-1970s, the government supported health service was available mainly in the urban areas. The services were more curative than preventive in nature. The government of Bangladesh accepted the primary health care concept as national health objective in 1978. Since then, the health care system was reoriented to provide essential health care to the general mass. In fact, the government significantly increased the funding of health sectors from early 1980s. The facilities include Maternal and Child Welfare Centre in urban and sub-urban areas, Upazila Health Complex at Upazila level and the Family Welfare Centre at union level. The government has also made primary health service facilities available at Rural Dispensaries and Satellite Clinics. In Matlab town, the government runs a 31-bed free general hospital along with few union-level health facilities. In addition, the government has been promoting oral rehydration therapy for diarrhoea management, and the immunization program against six major childhood diseases [31]. In fact, immunization against childhood diseases is mainly delivered through satellite clinics (since mid-1980s) while family planning services are delivered at the doorstep until recently. These services, however, less intensive than those services in the ICDDR,B-service area.

As health services are added gradually in both the ICDDR,B-service and govt-service areas, four birth cohorts (1983–85, 1988–90, 1993–95 and 1998–00) under study benefited differently from these interventions. For example, measles vaccination to all children started in 1982 in two Blocks of the ICDDR,B-service area while such vaccination started in 1985 in other two Blocks. In the govt-service area, immunization program was initiated in late-1980s and slowly gained popularity and in recent years the coverage rate is about 70%. So, children in the earlier cohorts benefited less from interventions than (immunization coverage differ) those in the recent cohort.

### Data

The study used HDSS data from both the ICDDR,B-service and govt-service areas. Four population-based birth cohorts were selected for the study. The HDSS system registered 21,280 births for 1983–85, 20,370 births for 1988–90, 16,925 births for 1993–95 and 17,102 births for 1998–00. These births were subsequently followed for five years for death and out-migration to ascertain survival and migration status respectively.

The socio-economic status is defined here in term of assets, rather than income or consumption. The asset information was collected through the household questionnaire administered during the censuses of 1982 and 1996. These questions include ownership of a number of consumer items (radio, watch, etc), dwelling characteristics (wall and roof material), type of drinking water and toilet facilities, however, more consumer items were collected in 1996 than in 1982 census.

Household economic status is measured in this study by constructing a wealth index using asset ownership as it was validated [32]. Each household asset for which all information collected was assigned a weight or factor score generated through principal components analysis. The resulting asset scores were standardized in relation to a standard normal distribution with a mean of zero and a standard deviation of one. Each household was assigned a standardized score for each asset, where the score differed depending on whether or not the household owned that asset. These scores were summed according to household and individuals were ranked according to the total score of the household in which they resided. The household were then divided into quintiles. Birth cohort-1983–85 and birth cohort-1988–90 were matched with the household asset score of 1982 and birth cohort-1993–95 and birth cohort-1998–00 were matched with household asset score of 1996^b ^[Appendix A].

Based on asset quintiles, two indicators of inequality are created: poor-rich ratio and concentration index [33]. The ratio (poor-rich) does not provide any information about the middle three quintiles but provides a magnitude of differences between the poorest and the richest quintiles of the population. On the other hand, concentration index measures the extent to which a particular health status variable is distributed across all five asset quintiles- that is the concentration of inequality. The concentration indices take the values between -1 and 1. The closer is the index to zero for any one health indicator, the less concentrated is the wealth inequality for that indicator; conversely, the further away is the index from zero, the greater is the inequality. A negative value indicates disproportionate concentration of the variable among the poor while a positive value indicates that the poor are getting less than would be expected had the distribution been equitable.

Life table mortality rates were calculated for neonatal, infant and child mortality.

## Results

Table 1 shows mortality rates by cohort, age and study area. For the initial cohort (1983–85), under-five mortality was 25% higher in the govt-service than the ICDDR,B-service area. Such area difference remained same (25%) for the second cohort (1988–90) but increased to 47% for the 3^rd ^cohort (1993–95) and then reduced to 30% for the 4^th ^cohort (1998–00). Increase in area difference of under-five mortality for 3^rd ^cohort is mainly due to increase in difference of neonatal mortality (35.2% vs 55.7%). As under-five year mortality difference in these two areas is mainly due to difference of neonatal mortality and causes of neonatal mortality differ from those of child mortality, subsequent analyses are done separately for neonate, infant and child.

**Table 1:** Mortality rates (per 1,000 live-births) by cohort and age, ICDDR,B-service and govt-service areas

Cohort/ratio	ICDDR,B-service				Govt-service			
	Neo-natal	Infant	1–4 yrs	Under-5	Neo-natal	Infant	1–4 yrs	Under-5

i: 1983–85	56.4	99.8	40.8	140.6	68.4	116.9	58.7	175.6
ii: 1988–90	45.4	74.3	22.9	97.2	53.6	89.4	32.0	121.4
iii:1993–95	35.2	58.9	17.7	76.6	55.7	85.7	27.0	112.7
iv:1988–00	31.3	47.6	11.5	59.1	41.2	60.0	17.8	77.8
ii:i	0.80	0.74	0.56	0.69	0.78	0.76	0.54	0.69
iii:i	0.62	0.59	0.43	0.54	0.81	0.73	0.46	0.64
iv:i	0.55	0.48	0.28	0.42	0.60	0.51	0.30	0.44

In both the areas, poor-rich ratio of neonatal mortality has widened overtime: it increased in the ICDDR,B-service from 1.10 in 1983–85 to 1.45 in 1988–90 and then declined slightly to 1.41 in 1993–95 and increased again to 2.00 in 1998–00 area compared to 1.04 to 1.29 to 1.53 and to 1.63 respectively in the govt-service area (Table 2). The 'difference of difference' assessment of poor-rich mortality ratios between the initial cohort (1983–85) and 4^th ^cohort (1998–00) show that it increased 81.8% in the ICDDR,B-service and 56.7% in the govt-service area (Table 3). A similar pattern is also shown with the concentration index, absolute value increased 185.3% in the ICDDR,B-service area (-0.068 to -0.194) while it increased 76.5% in the govt-service area (-0.051 to 0.090). Almost a similar pattern is followed for infant mortality because most death occurred during neonatal than post-neonatal period.

**Table 2:** Mortality rates (per 1,000 live-births) for different age by wealth index and cohort, ICDDR,B-service and govt-service areas

Wealth index	ICDDR,B-service				Govt-service			
	1983–85	1988–90	1993–95	1998–00	1983–85	1988–90	1993–95	1998–00

**Neonatal**								
Poorest	54.1	51.6	38.0	43.7	71.6	66.8	66.1	43.6
Richest	49.1	35.5	27.0	21.9	68.8	51.6	43.1	26.8
Poor:rich	1.10	1.45	1.41	2.00	1.04	1.29	1.53	1.63
**Infant**								
Poorest	100.3	86.6	67.3	59.6	132.9	105.0	97.9	74.6
Richest	92.2	57.6	43.9	30.2	106.0	81.1	63.6	38.0
Poor:rich	1.09	1.50	1.53	1.97	1.25	1.29	1.54	1.96
**1–4 years**								
Poorest	52.0	36.5	21.1	12.9	80.3	52.0	40.2	25.3
Richest	22.7	16.8	10.4	10.4	46.6	19.5	18.2	5.8
Poor:rich	2.29	2.17	2.03	1.13	1.72	2.67	2.12	4.36
**Under-5**								
Poorest	152.3	123.1	88.4	72.5	213.2	157.0	138.1	99.9
Richest	114.9	74.4	54.3	41.6	152.6	100.6	81.9	43.8
Poor:rich	1.33	1.65	1.63	1.74	1.40	1.56	1.69	2.28

**Table 3:** Changes in socioeconomic (poor:rich) inequalities in mortality for different ages

Study area/mortality	Poor:Rich					Concentration Index				
	1983–5 (1)	1988–0 (2)	1993–5 (3)	1998–0 (4)	(4):(1) (% diff.)	1983–5 (6)	1988–0 (7)	1993–5 (8)	1998–0 (9)	(9):(6) (% diff.)

**Neonatal:**										
1:ICDDR,B	1.10	1.45	1.41	2.00	+81.8%	-0.068	-0.136	-0.088	-0.194	185.3%
2:Government	1.04	1.29	1.53	1.63	+56.7%	-0.051	-0.099	-0.033	-0.090	76.5%
Row 1-Row 2					**+25.1**					
**Infant:**										
1:ICDDR,B	1.09	1.50	1.53	1.97	+80.7%	-0.081	-0.146	-0.107	-0.198	144.4%
2:Government	1.25	1.29	1.54	1.96	+56.8%	-0.087	-0.099	-0.032	-0.199	128.7%
Row 1-Row 2					**+23.9**					
**1–4 years**										
1:ICDDR,B	2.29	2.17	2.03	1.13	-50.6%	-0.197	-0.216	-0.152	-0.090	-54.3%
2:Government	1.72	2.67	2.12	4.36	+153.5%	-0.185	-0.241	-0.143	-0.208	12.4%
Row 1-Row 2					**-204.1**					
**Under-5**										
1:ICDDR,B	1.33	1.65	1.63	1.74	+30.8%	-0.114	-0.161	-0.118	-0.176	54.4%
2:Government	1.40	1.56	1.69	2.28	+116.3%	-0.117	-0.136	-0.057	-0.138	17.9%
Row 1-Row 2					**-85.5**					

Figures in bold preceded by a plus sign denote increases in inequality that were larger in the ICDDR,B area than in the government area. Minus signs before bold figures refer to inequality increases that were smaller (or decreases that were larger) in the ICDDR,B area

On the other hand, poor-rich ratio of child mortality decreased over time in one area but increased in other: it decreased in the ICDDR,B-service area from 2.29 in 1983–85 to 2.17 in 1988–90 to 2.03 in 1993–95 and to 1.13 in 1998–00 compared to 1.72 to 2.67 but declined to 2.12 and then increased again to 4.36 respectively in the govt-service area. The 'difference of difference' assessment of poor-rich ratios between the initial cohort (1983–85) and 4^th ^cohort (1998–00) show that mortality declined 50.6% in the ICDDR,B-service but increased to 153.5% in the govt-service area (Table 3). A similar pattern is also shown with the concentration index, absolute value decreased 54.3% in the ICDDR,B-service (-0.197 to -0.090) but it increased 12.4% in the govt-service area (-0.185 to -0.208).

For under-five mortality, poor-rich ratio has widened over time in both the areas: it increased in the ICDDR,B-service area from 1.33 in 1983–85 to 1.65 in 1988–90 and then reduced slightly to 1.63 in 1993–95 and increased again to 1.74 in 1998–00 compared to 1.40 to 1.56 to 1.69 and to 2.28 respectively in the govt-service area. The 'difference of difference' assessment of poor-rich ratios between the initial cohort (1983–85) and 4^th ^cohort (1998–00) show that mortality increased 30.8% in the ICDDR,B-service area while it increased to 116.3% in the govt-service area. A similar pattern is also shown with the concentration index, absolute value increased 54.4% in the ICDDR,B-service (0.114 to 0.176) and it increased 17.9% in the govt-service area (0.117 to 0.138).

## Discussion

Before interpreting results, the following points should be kept in mind. Measuring the impact of health intervention on time trend data is complex because some interventions might reach the poor after a certain period and can reduce the poor-rich difference while at the same time a new intervention that might be used more by rich than the poor and might have increased the poor-rich difference^c ^[Appendix A]. Moreover, aggregate level data might show a different pattern of inequality than disaggregated data.

For neonatal mortality, socioeconomic inequalities increased over time in both the areas but it increased more in the ICDDR,B-service area. In the recent cohort, neonates of rich household survived more in the ICDDR,B-service than in the govt-service area and it could be due to nature of health intervention. In fact, the community based maternity care programme was initiated in the ICDDR,B-service area in late-1980s and it was changed to facility-based program in 1996^d ^[Appendix A]. This intervention had impact in reducing the direct obstetric mortality [35] and these services were used more by rich than the poor [36], thus have resulted such poor-rich difference in neonatal survival.

On the other hand, socioeconomic inequalities of child mortality decreased considerably in the ICDDR,B-service area while it had increased in the govt-service area. Narrowing socioeconomic inequalities of child mortality in the ICDDR,B-service area could be due to high coverage of immunization (almost universal) that resulted similar survival. On the other hand, immunization coverage of the govt-service area is relatively low compared to ICDDR,B-service area (60% vs 95%) and is likely to be used more by the rich than the poor that might have resulted increased poor-rich ratio.

Our analysis support to some extent the inverse care law which states that 'the new intervention will tend to increase the inequality since they will initially reach those who are already better off' [27], as documented in case of neonates in our study. The same study also reported that 'the inequality will reduce while wealthy reach a level when little progress can be made', and this is observed for child mortality in the ICDDR,B-service area. Moreover, studies documented in Bangladesh that some interventions are more equitable (for example, immunization program) than others (for example, use of antenatal care and delivery service) and such inequality pattern depends on the mode of service delivery as well as its level of utilization.

The findings of the study are encouraging because mortality is declining in both the areas and there is evidence that the socioeconomic inequality might decline in future as documented in the ICDDR,B-service area for child mortality. But health intervention in the ICDDR,B-service area is so intensive that it might not be possible to replicate it at the national level, however, can guide the policy planners in formulating appropriate health policy.

## Authors' contributions

AR carried out the study design, data management and analysis, and drafted and revised the manuscript. PKS and DRG participated in designing the study and revising the manuscript. All the authors' approved the final manuscript.

## Appendix A

^a^There were 17 death cases were missing from the Muhuri's data file and affected the result.

^b^Assets were available only for 1982 and 1996 (collected during the socioeconomic survey). So, birth cohorts were matched with the asset scores of the closest one.

^c^Users of an asset index should be aware that choice of assets influences the outcomes observed. So, researchers should carefully select the items they include in the index [34].

^d^Beginning in 1996, facility-based maternity care service was introduced in ICDDR,B-service area. The sub-centres were equipped to provide basic emergency obstetric care for the catchment area and are posted with a trained nurse-midwife along with a paramedic. These nurse midwives and paramedics have been trained to provide antenatal care, treat minor complications, conduct normal deliveries and refer cases with complications to Matlab hospital.
